# Evaluating Tidal Marsh Sustainability in the Face of Sea-Level Rise: A Hybrid Modeling Approach Applied to San Francisco Bay

**DOI:** 10.1371/journal.pone.0027388

**Published:** 2011-11-16

**Authors:** Diana Stralberg, Matthew Brennan, John C. Callaway, Julian K. Wood, Lisa M. Schile, Dennis Jongsomjit, Maggi Kelly, V. Thomas Parker, Stephen Crooks

**Affiliations:** 1 Climate Change and Informatics Group, PRBO Conservation Science, Petaluma, California, United States of America; 2 Department of Biological Sciences, University of Alberta, Edmonton, Canada; 3 Estuaries and Wetlands Team, ESA PWA, San Francisco, California, United States of America; 4 Department of Environmental Science, University of San Francisco, San Francisco, California, United States of America; 5 Department of Environmental Science, Policy and Management, University of California at Berkeley, Berkeley, California, United States of America; 6 Department of Biology, San Francisco State University, San Francisco, California, United States of America; University of Western Australia, Australia

## Abstract

**Background:**

Tidal marshes will be threatened by increasing rates of sea-level rise (SLR) over the next century. Managers seek guidance on whether existing and restored marshes will be resilient under a range of potential future conditions, and on prioritizing marsh restoration and conservation activities.

**Methodology:**

Building upon established models, we developed a hybrid approach that involves a mechanistic treatment of marsh accretion dynamics and incorporates spatial variation at a scale relevant for conservation and restoration decision-making. We applied this model to San Francisco Bay, using best-available elevation data and estimates of sediment supply and organic matter accumulation developed for 15 Bay subregions. Accretion models were run over 100 years for 70 combinations of starting elevation, mineral sediment, organic matter, and SLR assumptions. Results were applied spatially to evaluate eight Bay-wide climate change scenarios.

**Principal Findings:**

Model results indicated that under a high rate of SLR (1.65 m/century), short-term restoration of diked subtidal baylands to mid marsh elevations (−0.2 m MHHW) could be achieved over the next century with sediment concentrations greater than 200 mg/L. However, suspended sediment concentrations greater than 300 mg/L would be required for 100-year mid marsh sustainability (i.e., no elevation loss). Organic matter accumulation had minimal impacts on this threshold. Bay-wide projections of marsh habitat area varied substantially, depending primarily on SLR and sediment assumptions. Across all scenarios, however, the model projected a shift in the mix of intertidal habitats, with a loss of high marsh and gains in low marsh and mudflats.

**Conclusions/Significance:**

Results suggest a bleak prognosis for long-term natural tidal marsh sustainability under a high-SLR scenario. To minimize marsh loss, we recommend conserving adjacent uplands for marsh migration, redistributing dredged sediment to raise elevations, and concentrating restoration efforts in sediment-rich areas. To assist land managers, we developed a web-based decision support tool (www.prbo.org/sfbayslr).

## Introduction

Projections of sea-level rise (SLR) range from 18 cm to nearly 2 m over the next century [1], [2] (and recent assessments suggest that as much as 5 m could be possible [3]), making low-lying coastal zones particularly vulnerable to climate change. The primary threats of SLR are well known: exacerbated beach and shoreline erosion, and inundation of critical infrastructure and coastal wetlands [4]–[6]. Uncertainty about how dynamic ecosystems such as coastal and estuarine tidal marshes (hereafter “tidal marshes”) may respond to different aspects of climate change has prompted a large body of research exploring potential tidal marsh responses to increased rates of SLR [7]–[9], as well as increased temperature [10], salinity [11], and CO_2_ concentrations [12].

Tidal marshes provide high-value ecosystem services such as water filtration, flood abatement, protection for infrastructure, and carbon sequestration [13]–[15]. They also have high ecological value, supporting a large number of specialized and endemic species [16], [17] and have already experienced dramatic historical declines in area and hydrologic integrity [18]. The sensitivity of tidal marshes to increased rates of SLR will vary depending upon factors such as mineral sediment supply [19], vegetation productivity [7], rates of subsidence or uplift [20], changes in storm frequency and intensity [21], and availability of uplands suitable for marsh migration [22]. Estuarine systems with low sediment inputs and high rates of subsidence such as the Mississippi River Delta have already experienced substantial marsh loss due to relative SLR (i.e., including the influence of subsidence) [23], while sediment-rich systems such as parts of San Francisco Bay have demonstrated resilience to rapid rates of relative SLR [24], [25].

Tidal marshes are dynamic ecosystems that occupy a relatively narrow band of elevation, governed primarily by vegetation tolerance of tidal inundation, along with other factors, including hydroperiod, sediment supply, and biological dynamics [7], [8], [26], [27]. With adequate sediment supply, the marsh plain builds to an elevation high within the tidal frame, typically around mean higher high water (MHHW) under semidiurnal tides [28]. At higher elevations, reduced tidal inundation curtails building processes through reduced mineral sediment supply and oxidation of soil organic material. At lower elevations, increased flooding frequency and duration increase mineral sedimentation and therefore enhance marsh building. In addition, vegetation plays an important role in trapping sediment and contributing organic material through above- and below-ground growth [29], [30], with additional potential feedbacks between elevation and plant dynamics [7].

Under conditions where rates of SLR exceed marsh building processes the marsh plain falls in elevation relative to the tidal frame. A new steady state may be achieved, reflecting increased sedimentation at lower elevations that balances increased SLR. Alternatively, if supply of sediment is inadequate to keep pace with SLR, the marsh plain will continue to fall relative to sea level, eventually to an elevation where vegetation cannot tolerate the prolonged inundation, and the marsh will transition to a mudflat [9], [31]. When topographically suitable uplands are lacking or located behind levees (as in most urbanized estuaries), marshes will not be able to migrate landward as they have done historically, resulting in marsh loss.

Previous research has shown a positive relationship between local rates of relative SLR and rates of sediment accretion [24], [25], [32]. However, increased sediment accretion in response to SLR is limited by mineral sediment inputs as well as plant growth and organic material accumulation, which may decrease in response to increases in salinity resulting from SLR and changes in precipitation regimes [11]. Measured rates of sediment accretion in tidal marshes have varied from 1 to 15 mm/yr, with the highest rates recorded in regions with very high rates of relative SLR driven by local subsidence, e.g., parts of Chesapeake Bay, the Mississippi River Delta, and other large delta systems [33]–[36]. However, the likelihood that tidal marshes can keep pace with high rates of SLR appears to diminish rapidly if rates of relative SLR are more than 10 mm/yr or increase rapidly [37], [38].

With hundreds of millions of dollars invested in tidal marsh restoration and conservation, management strategies need to clearly identify and integrate thresholds and sensitivities of mineral sediment supply, organic accumulation rates, and starting elevation for marsh sustainability under various climate change scenarios. The long-term persistence of these habitats also depends on our ability to identify and protect areas where marshes can move upland as sea level rises and to identify barriers to that movement, such as levees. Conservation planners need to know where in the landscape tidal marshes will have the greatest long-term sustainability and how to prioritize restoration activities. To address these problems, spatially explicit projections of tidal marsh sustainability and restoration potential are needed at the estuary scale.

Many modeling approaches have been implemented and have improved our understanding of marsh responses to increased rates of SLR [39]. The challenge in developing models for tidal marshes is to combine realistic local processes of sediment feedbacks with broader scale (i.e., estuary-wide) spatial dynamics. Many models have accurately represented realistic local processes, focusing on mineral and/or organic material dynamics [7], [31], [40]. Most of these models lack spatial variability, although recently-developed geomorphic models also incorporate channel dynamics and erosion across the marsh plain surface [41], [42]. Other models, such as SLAMM (sea level affecting marshes model), have focused on broad-scale spatial patterns but have not realistically modeled feedbacks of elevation on sediment dynamics or other critical local processes [43], [44]. Combining high resolution process-based models with broad-scale spatial modeling that includes hydrodynamics would be ideal; however, this is very computer intensive and is subject to potential accumulation of errors across multiple time steps. Although estuary-wide mechanistic approaches are being developed, the application of this sort of model is currently not practical.

Given the increasing interest among resource managers in spatially-explicit, estuary-wide assessments of potential SLR impacts on tidal marshes, we developed a hybrid method that involves a realistic, mechanistic treatment of marsh accretion dynamics and incorporates spatial variation across an estuary. Our approach is simple, transparent, and easily transferable and updatable, such that results can be readily accessible to land managers. At the core is a process-based model of point-based mineral accumulation based on Krone's [45] model called Marsh98 [9], [46], which includes feedbacks between elevation and sediment inputs and incorporates constant rates of organic accumulation. We extended the point-based predictions to develop spatially-explicit projections of marsh sustainability based on current marsh elevation at the 5-m pixel level, and characterization of mineral (suspended sediment concentrations) and organic (relative plant productivity) inputs at the level of biogeomorphic subregions. While this approach lacks the hydrodynamic component to spatially transport sediment, it still allows for the evaluation of realistic process-based accretion dynamics and is feasible to apply across an entire estuary, over long time frames, and across multiple scenarios. It is of particular interest in the San Francisco Bay, California, USA (hereafter “Bay”), where, since European settlement, more than 90% of tidal marshes across the Bay have been destroyed or altered, primarily through agricultural and urban land development [47], [48]. Many of the Bay's remaining marshes are adjacent to developed urban areas with minimal or no natural upland buffer zones. The large-scale loss of Bay wetlands has caused dramatic functional changes to the region over the last 150 years, affecting endangered and endemic species. Furthermore, over $60 billion in infrastructure is at risk of inundation under high rates of SLR [49]; some of this loss could be prevented with tidal marsh restoration. Thus, there is considerable interest to maintain the integrity of current tidal marshes and facilitate restoration of diked baylands throughout the Bay [50].

Herein, we used our modeling approach to explore the sustainability of tidal marshes under a range of SLR and sediment availability conditions, using San Francisco Bay as a case study. In doing so, we sought to answer the following key questions: (1) What are the thresholds and sensitivities for marsh sustainability in terms of mineral sediment supply, organic material contribution, SLR rates, and starting elevations? (2) How is the Bay-wide area and composition of intertidal habitats likely to change under varying projections for SLR and sediment availability? (3) How much space exists for new marshes to form, and how much habitat may be expected under these different scenarios? Our goal was also to deliver results to land managers in an easily accessible and interactive web-based map tool, to support conservation planning and restoration activities.

Specifically, we evaluated eight scenarios for bay-wide change over the next century, intended to capture low and high levels of potential outcomes based on a combination of factors:

- Two subregion-specific levels of suspended sediment concentration (SSC)
- Two subregion-specific levels of organic material (OM) accumulation
- Two rates of SLR (0.5 and 1.65 m/century)

We evaluated these eight scenarios over the range of actual starting elevations and estimated levels of SSC and OM accumulation found throughout the Bay.

## Materials and Methods

### Study area

**Figure 1:**
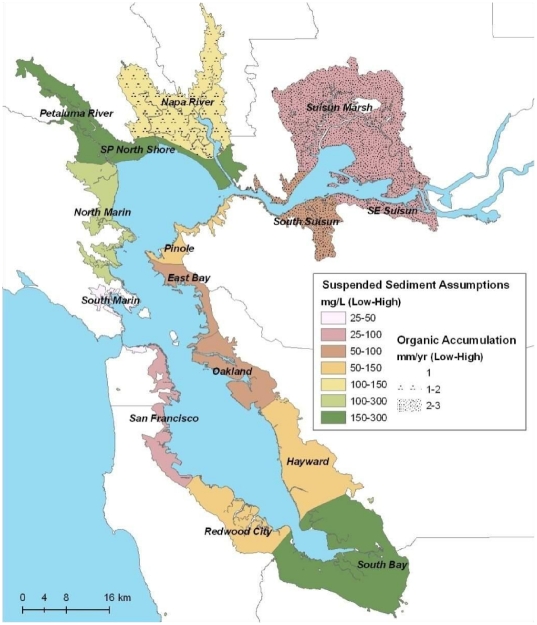
Biogeomorphic subregions within San Francisco Bay study area and assumptions about suspended sediment concentrations and organic matter accretion rates for climate change scenarios.

Our study area within the San Francisco Bay, which is characterized by a mixed semi-diurnal tide cycle, includes salt water and brackish tidal marshes west of the confluence of the San Joaquin and Sacramento rivers (Fig. 1). The area has a Mediterranean-type climate, with warm, dry summers and rainy, cool winters [51]. Rain and runoff from snow pack of the Sierra Nevada mountains create lower salinity conditions in the Bay during the winter and spring, with significantly reduced freshwater influx and higher salinity during the summer and fall [52]. Plant species richness and productivity are greater in lower salinity tidal marshes [28], [53].

Bay tidal marshes owe their early development to changes in sea level. During the last glacial event, San Francisco Bay was a river valley. By about 5,000 years before present, sea level had risen to an elevation adequate to flood the Bay, creating conditions for fringing tidal wetlands [54], [55]. These wetlands continued to build and transgress landwards over subsequent millennia. Seasonal flows of the Sacramento River, as well as from local catchments, brought sediment to the Bay, maintaining expansive marshes and mudflats. Tidal marshes and mudflats continued to expand through the 1800 s, when hydraulic mining activities in the Sierra Nevada foothills deposited considerable sediments in the Bay, estimated to be an order of magnitude larger than pre-mining conditions [55], [56].

During the 20th century, filling and levee building activities reduced tidal marshes to less than 10% of their original 220,000 ha [28] although approximately 5,000 ha have since been regained through restoration efforts [57]. Upstream activities such as dams, water diversions, riverbank protection, and altered land use limited the downstream delivery of sediment and caused erosion of subtidal habitats [58]. Since 1999, a substantial decrease in suspended sediment has been observed at long-term deepwater monitoring stations [59]. This step change is attributed to the flushing of the hydraulic mining pulse from the estuary and limitations on downstream delivery [60], [61].

The current Bay wetland landscape west of the Sacramento/San Joaquin River delta is an intricate mosaic of natural and restored tidal marshes intermixed with diked baylands. Tidal marshes line the bay and river margins and, in most cases, abut levees along urban and agricultural land. We defined the bayward limits of our study area based on the mapped edge between tidal marsh and mudflat habitats according to the San Francisco Estuary Institute's EcoAtlas (http://www.sfei.org/ecoatlas/index.html) and used the USGS national elevation dataset (NED) to delineate upland boundaries. The upper limit was defined as the15.2 m (50-ft.) elevation contour line plus a 100-m horizontal buffer to account for error in the NED, resulting in a total study area of just over 186,000 ha. Mapping of study area boundaries and subregions was performed in ArcGIS 9.3.1 (ESRI, Redlands, CA, USA).

### Biogeomorphic subregions

Suspended sediment concentrations (SSC) differ throughout the Bay because of variations in wave conditions, proximity to mudflats, bathymetric convergence zones, and river inputs. These subregional differences help define the morphology, extent, and resilience to SLR of Bay tidal marshes. In addition, marshes with high rates of organic matter (OM) production have been observed to accrete at faster rates than marshes composed primarily of inorganic sediments [7], [40]. Marshes associated with the highest OM accumulation rates are typically found in brackish and freshwater environments.

In light of this spatial variation, we separated the Bay into 15 biogeomorphic subregions (ranging in area from 2,123 to 34,605 ha) based on sediment and salinity characteristics (Fig. 1). Each subregion was categorized according to “low” and “high” estimates of SSC and OM for that subregion, based on information described in the following sections and summarized in Table S1. These subregion-specific “low” and “high” values were used to explore scenarios of high/low SSC and OM.

### Accretion model

Marsh accretion (the vertical accumulation of sediment mineral and organic material) was estimated using the Marsh98 model, which has been used widely to examine marsh response to SLR across San Francisco Bay [9]. The Marsh98 model is based on the mass balance calculations described by Krone [45]. This model assumes that the elevation of a marsh surface increases at a rate that depends on the (1) availability of suspended sediment and (2) depth and periods of inundation by high tides. Marsh98 implements these processes by calculating the amount of suspended sediment that deposits during each period of tidal inundation and sums that amount of deposition over the period of record. OM was added directly to the bed elevation at each time step at a constant rate (see below for details). Marsh98 was implemented in the Fortran programming language, and multiple runs were executed using MatLab v.2010b (MathWorks Inc., Natick, MA).

Modeling was conducted relative to the tidal datum of mean lower low water (MLLW) and converted to mean higher high water (MHHW) based on a 1.8-m tide range. The tidal boundary condition used for all model runs was a repeated tidal month that has statistical characteristics representative of the observed tides at the mouth of San Francisco Bay and in the North and Central Bays. However, the tides are naturally amplified in the South Bay such that the tide range increases by approximately 50% at the far southern end of the Bay. The tide range diminishes in Suisun Bay and eastward into the Sacramento/San Joaquin Delta.

Given the spatially-varying tide range, a sensitivity analysis was conducted testing the impact of a larger tide range on the marsh accretion rates and elevation. For cases with moderate to high SSC as are typically found in the South Bay, simulations run with a tide range of 2.8 m predicted marsh surface elevations after a century that were at most 0.2 m lower relative to MHHW than simulations using a 1.8-m tide range (although overall accretion was higher). In relative terms, this difference is less than 5% of the total predicted accretion for all cases. Thus, we used a single tidal range (1.8 m) to simplify the analysis.

### Model input parameters

To address the range of conditions across the Bay, as well as climate change uncertainty, we considered seven SSC levels, three OM accumulation rates (except for scenarios with subtidal initial elevations, which included no OM), two rates of SLR, and three initial bed elevations, for a total of 70 model runs (90 possible–20 subtidal/OM combinations not considered). Various combinations of these 70 model runs were combined at the subregion level and interpolated to a range of starting elevations to generate six bay-wide spatial change scenarios.

#### Initial bed elevation

Two of the initial bed elevations evaluated span the range of regularly inundated vegetated marsh, the lower of which was based on the colonization elevation for vegetation (low marsh), assumed to be −0.5 m MHHW (mean tide level plus 0.3 m or 1.3 m MLLW) [62]. The higher initial bed elevation was based on the standard marsh plain (mid marsh) elevation around 0 m MHHW (1.8 m MLLW). The third initial bed elevation at −2.4 MHHW (0.6 m below MLLW) was used to predict the bed elevation trajectory for marsh development from subtidal conditions.

### Rate of SLR

**Figure 2:**
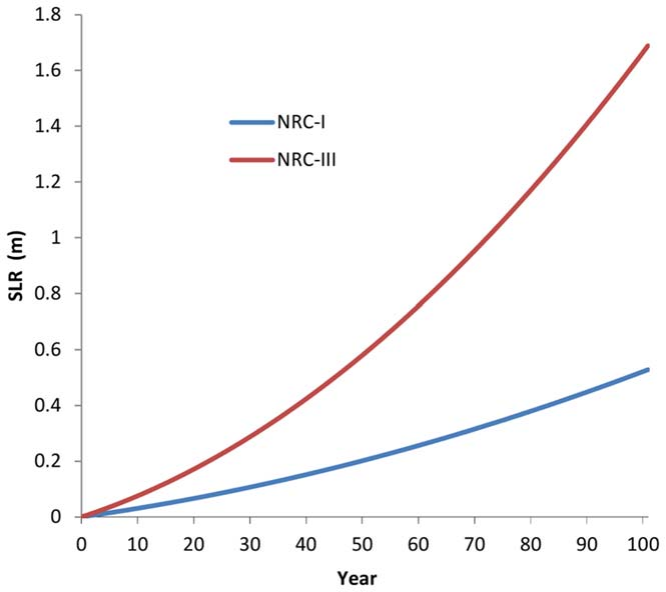
High (NRC-III) and low (NRC-III) sea-level rise trajectories used for climate change scenarios. Year 0 represents 2010 and year 100 represents 2110.

We chose two nonlinear SLR scenarios based on the guidance provided by the US Army Corps of Engineers [63], which recommends scenarios modifying curves proposed by the National Research Council to extrapolate intermediate and high SLR scenarios (“NRC-I” and “NRC-III”, respectively). These scenarios project 0.52 m and 1.65 m of SLR over the next century (2010 to 2110) with most of this change occurring within the second half of the century (Fig. 2). The high-end rates are similar to recent estimates [1], [64], and to the draft State of California planning guidelines, which recommend planning for 0.41 m of rise in the next 50 years and 1.4 m in the next 100 years [65].

#### Suspended sediment concentration

To represent the range of observed SSC, we modeled seven different concentrations: 25, 50, 100, 150, 200, 250, and 300 mg/L. Although observations of SSC within Bay tidal marshes are limited, several deepwater (major channel and open bay) data sources helped inform this range. The first four values are representative of observed SSC along the deepwater channel [66]. SSC at the bay-marsh boundary is thought to be higher because of wave resuspension over nearby mudflats [67]. For tributaries entering the North Bay, Ganju et al. [68] corroborate the concentrations at the high end of our range. A second line of evidence for the high SSC values comes from calibrations of the Marsh98 model to observed rates of bed elevation change at several restoration sites around the Bay [46].

#### Organic material

Based on data from over 30 dated sediment cores (^137^Cs and ^210^Pb) from multiple sites across the Bay (Callaway, unpublished data), we modeled OM accretion using constant rates of 1, 2, and 3 mm/yr for the scenarios with initial bed elevations in the vegetated marsh regime. For the scenarios with subtidal initial bed elevations, no OM accretion was included. As a sensitivity analysis, for one test run based on high SSC (150 mg/L) and high SLR, we also ran the model in two stages, adding OM from the point at which the bed elevation reached the vegetation colonization elevation; differences in final elevations were negligible.

### Elevation and tidal range mapping

A seamless 5-m elevation grid for the study area was developed based on best available data sources (Figure S1). LiDAR elevation data were available for most of our study area and were used wherever possible. Approximately 4,300 ha of diked subtidal lands (including several former and active salt ponds) were inundated with water and thus not captured by elevation mapping efforts. All datasets were converted to the NAVD88 vertical datum (m) and resampled to a 5-m×5-m grid-cell resolution. While a comprehensive accuracy assessment was not possible, we used available real-time kinetic GPS data (horizontal accuracy: ±1–2 cm; vertical accuracy: ±2–3 cm) from four North Bay study sites to investigate potential systematic biases in the datasets. Due to obvious vegetation biases in two of these sites in Suisun Bay and the western Delta, where marsh vegetation (*Schoenoplectus* spp.) often forms particularly impenetrable mats, we used available vegetation data to develop correction factors for each general vegetation type (Table S2) and applied those correction factors throughout the relevant subregions based on available vegetation maps [69], [70].

NOAA tide gauge and benchmark data (http://tidesandcurrents.noaa.gov/) were used to convert NAVD88 elevations to a MHHW reference more suitable for cross-bay analysis of tidal marsh habitat due to variability in tidal range across the bay. We developed a second-order inverse distance-weighted interpolation of MHHW levels (relative to mean lower low water, MLLW) across our study area (n = 55 tide gauges). The same procedure was repeated for NAVD88 elevations at MLLW measured for n = 19 benchmark locations. The two resulting grids (100-m resolution) were applied as offsets to the resulting elevation grid, corrected for vegetation bias where data were available, resulting in a bay-wide estimate of elevation (m) with respect to MHHW. Simply stated: NAVD88 elevation + MLLW offset − MHHW offset = MHHW elevation.

### Spatial scenario development

Model outputs were linearly interpolated in 10-cm increments for starting elevations ranging from −3.7 to 1.7 m (relative to MHHW) such that for starting elevation *x* between starting elevations *y* and *z*, the future projection for a given time period *t* and scenario *s* was calculated as:
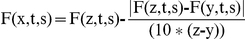



The lower bound for the interpolation was set at −4.0 m (MHHW), reflecting the lowest projected future elevation obtained from a model run starting at −2.4 m (MHHW). Elevations below this lower bound were assumed to remain constant (i.e., keep pace with SLR) across all scenarios and time steps. However, values are unreliable below −2.4 m due to the necessarily arbitrary lower limit for interpolation. The upper bound was set at 1.7 m (MHHW) for the high SLR scenario and 0.6 for the low SLR scenario, reflecting the area subjected to future tidal inundation. Elevations above the amount of SLR for a given scenario and time step were assumed to decrease by that amount (i.e., no accretion potential).

Interpolated model outputs were applied to a composite 5-m elevation grid for SF Bay, referenced to the MHHW tidal datum. Results for each combination of SSC, OM, and SLR assumptions were combined by geographic subregion to produce an individual scenario layer. For these scenarios, we assumed that wave- and current-induced bed shear stresses are minimal. Locations with significant wave exposure and/or tidal currents, which include much of the open bay margins, are unlikely to accrete above subtidal elevations. Thus we ignored current open bay and outboard mudflats, and restricted our analysis to areas currently landward of the marsh-mudflat boundary. We assumed that subsided (currently diked) potential restoration sites within our study area are not large enough to be subject to erosion at levels sufficient enough to prevent vegetation colonization. Indeed, no San Francisco Bay tidal marsh restoration sites have yet failed to vegetate [71].

### Analysis of marsh sustainability and restoration potential

Using the accretion model outputs for low marsh (−0.5 m MHHW) and mid marsh (0 m MHHW) starting elevations, we evaluated the potential for marsh sustainability over the next century (in 20-year increments) under each combination of SSC, OM, and SLR rate. The transition from low to mid marsh occurs approximately halfway between these elevations and mid marsh can persist at elevations lower than 0 m MHHW [62]. Thus a mid marsh area could lose elevation and still sustain marsh vegetation. However, because we were interested in the potential for a marsh to maintain its starting elevation our definition of marsh sustainability was zero elevation loss (rounded to the nearest 10 cm).

Due to the large number of planned restoration projects within subsided diked baylands, we also examined the minimum starting bed elevations required to achieve mid marsh elevations (−0.2 m to 0.1 m MHHW) over the next century in 20-year increments. This represents the potential to attain and maintain a vegetated marsh plain by restoring tidal action to currently diked (and generally subsided) areas. These calculations were based on elevation-interpolated model outputs to allow a broader range of starting elevations to be considered. Strictly speaking, we could not evaluate starting elevations lower than −2.4 m MHHW, the lowest bed elevation used in the accretion model runs. However, constantly-inundated subtidal elevations will accrete sediment very rapidly in the absence of significant erosional forces [46]. Thus, minimum starting bed elevations may be less than −2.4 m.

### Area calculations for restoration scenarios

We developed a polygon GIS layer representing all diked areas within our study area to distinguish existing from potential tidal marsh habitat. Diked areas were defined as those that were separated from regular tidal inundation by dikes, levees, or roads of any height and material; additional information on levee integrity was not readily available. The layer was modified from the EcoAtlas modern baylands layer (“diked baylands” category) based on levee lines supplied by the Pacific Institute (http://www.pacinst.org/reports/sea_level_rise/data/index.htm) and manual inspection of 1-m resolution natural color and color infrared Bay-wide aerial photography flown in 2006 and 2009 by the National Agriculture Imagery Program (http://www.fsa.usda.gov/FSA/apfoapp?area=home&subject=prog&topic=nai). We used a 2001 urban development layer from NOAA C-CAP (http://www.csc.noaa.gov/digitalcoast/data/ccapregional/) to identify developed areas not available for tidal marsh restoration.

Elevation projections were classified according to marsh type and summarized by subregion, scenario, and diked/developed status. Upland was defined as >0.3 m above MHHW; high marsh was defined as 0.2 to 0.3 m above MHHW; mid marsh as −0.2 to 0.1 m MHHW; low marsh as −0.5 to −0.3 m MHHW; mudflat as −1.8 to −0.6 m MHHW; and subtidal as anything below −1.8 MHHW (i.e., 0 m MLLW). We also compared restoration potential for areas of (1) high and (2) low-intermediate sediment availability within the currently diked areas. We used results from actual study area subregions grouped as follows: (1) high sediment availability (North Bay): Petaluma River, North Marin, San Pablo Bay North Shore; and (2) low-intermediate sediment availability (Central Bay): Redwood City, Hayward, San Francisco, Oakland, East Bay, Pinole, and South Marin (see Fig. 1 and Table S1). We selected these particular regions because they represent the range of sediment availability within the Bay, and because they have tide ranges similar to the 1.8-m value used in our accretion models.

### Web-based map viewer and decision support tool

To make our results easily accessible to land managers and decision-makers, a web-based map viewer and decision support tool was created that allows users to view projected changes in tidal marsh extent and location at varying spatial scales, over multiple time frames, and under various SLR, SSC, and OM scenarios [72]. Users can view maps of current and future marsh extent together with data overlays (diked areas, public lands, and urbanization) to assess restoration opportunities and impediments.

## Results

### Thresholds and sensitivities

#### Marsh sustainability

**Figure 3:**
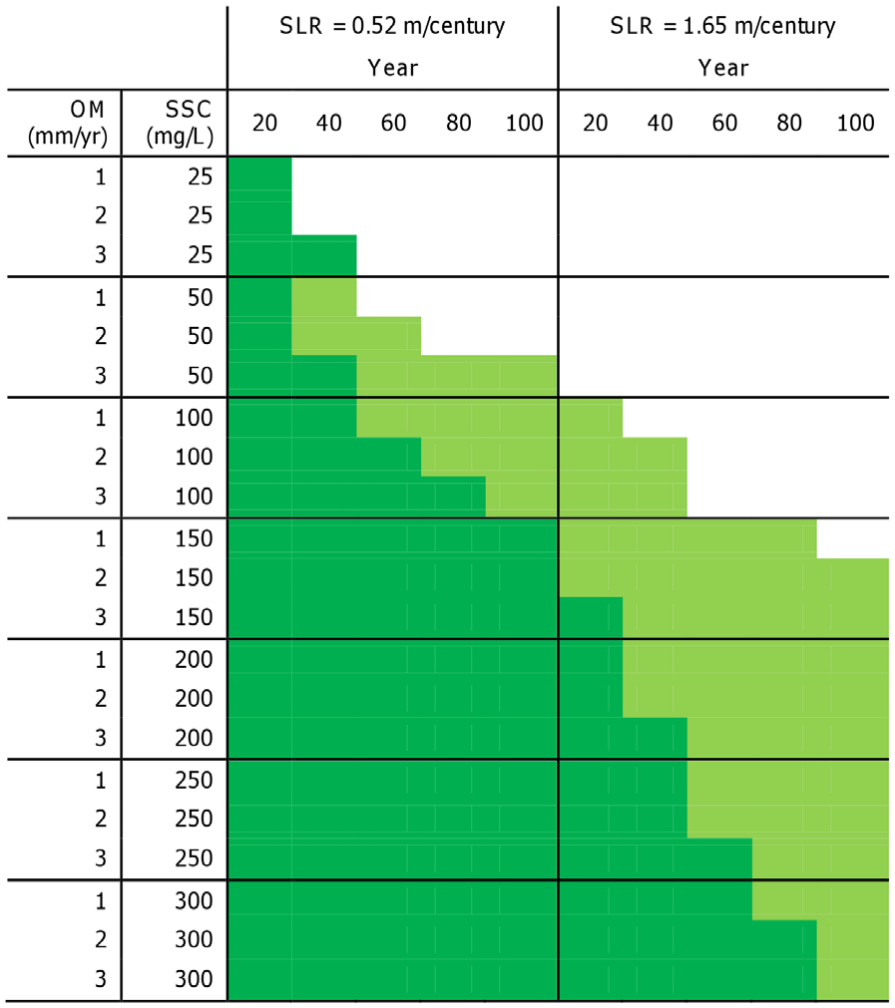
Sustainability (no elevation loss) of low marsh (light green) and mid marsh (dark green) areas under different sea-level rise scenarios, suspended sediment concentrations (SSC) and organic material contribution (OM). Blank cells represent no marsh sustainability.

According to accretion model outputs, marshes in areas with very low suspended sediment concentrations (25 mg/L) would not sustain their current elevation for more than 40 years under either SLR rate (Fig. 3). However, with high OM accumulation rates (3 mm/yr) and slightly higher SSC (50 mg/L), low marsh elevations would be sustained for up to 100 years under a low rate of SLR. Under a high SLR rate, marshes with 50 mg/L SSC would not be sustainable for 20 years regardless of OM (Fig. 3).

Under a low rate of SLR and intermediate SSC (100 mg/L), low marsh elevations would be sustained for 100 years, while mid marsh would last up to 80 years with high OM accumulation rates (Fig. 3). Under a high SLR rate and intermediate SSC, low marsh elevation loss would be expected within 40 years. With 150 mg/L, mid marsh sustainability throughout the next century was projected for a low SLR rate; only low marsh with at least 2 mm/year OM accumulation would be sustainable under a high rate of SLR. At 200, 250, and 300 mg/L, mid marsh was sustainable under a high rate of SLR for progressively longer periods of time (up to 80 years with 300 mg/L SSC), but not over the full 100-year period. Higher OM accumulation rates (2–3 mm/year) would not extend sustainability for more than a 20-year period.

#### Restoration potential and initial elevation

**Figure 4:**
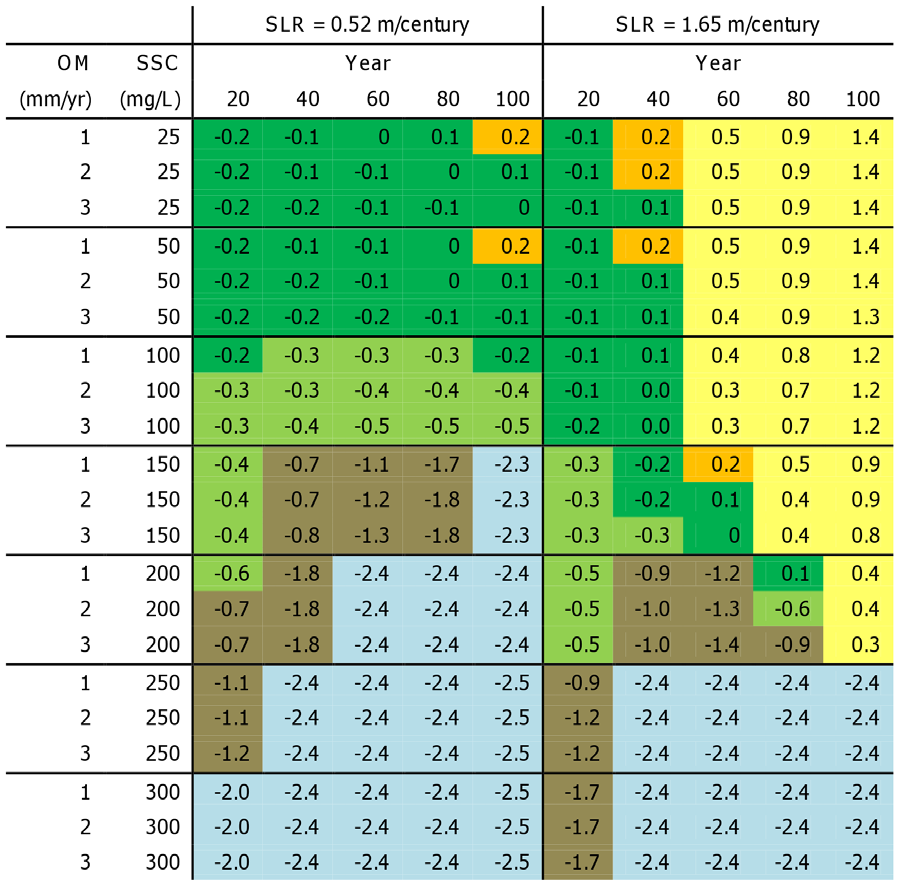
Minimum initial elevations with respect to MHHW needed to achieve mid marsh restoration (≥−0.2 m MHHW). Cells are color-coded to represent classification of initial conditions as follows: blue = subtidal, brown = mudflat, light green = low marsh, dark green = mid marsh, orange = high marsh, yellow = upland.

Under a low rate of SLR and high SSC (≥150 mg/L), our models show that mid marsh restoration (i.e., establishment and maintenance of a vegetated marsh plain) could be achieved over the next century with initial bed elevations at least as low as −2.4 m MHHW (i.e., subtidal) (Fig. 4). With very high SSC (300 mg/L), mid marsh habitat could be expected within 20 years at subtidal locations, while close to 100 years would be necessary with 150 mg/L. For low-intermediate sediment concentrations (≤100 mg/L), successful mid marsh restoration would be expected only from marsh starting elevations. Higher rates of organic accumulation (2–3 mm/yr) would allow somewhat lower starting elevations, but could not (by definition) make a difference of more than 20 cm per century.

Under a high rate of SLR, however, mid marsh restoration could only be achieved over a 100-year time period given starting elevations above MHHW (current upland areas), or very high sediment concentrations (Fig. 4). With very high SSC (250–300 mg/L), mid marsh habitat could be restored even in areas that are currently subtidal. At lower sediment concentrations, mid marsh could initially be restored from low- and mid-marsh starting elevations below MHHW but would not persist more than 80 years (40 years at very low SSC).

### Bay-wide habitat change

**Figure 5:**
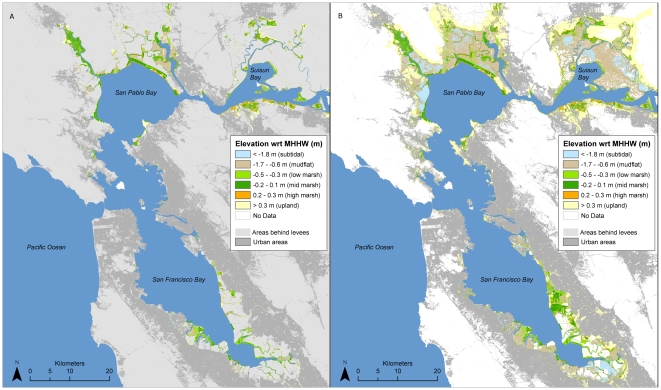
(A) Existing and (B) potential intertidal habitats in San Francisco Bay based on current mapped elevations. See Figure S1 for map of data sources.

Based on mapping of current elevations and barriers to tidal inundation, there are currently ∼2,500 ha of high marsh, 7,600 ha of mid marsh, and 3,000 ha of low marsh in San Francisco Bay (Table 1, Fig. 5a). An additional 7,500 ha of marsh (plus up to 4,300 ha of unmapped diked subtidal areas) could exist if existing dikes, levees, roads, and other barriers to tidal inundation were removed (Fig. 5b). 4,300 ha of potential tidal marsh are considered un-restorable due to urban development (Table 1). Below we detail projected changes over the next 100 years by habitat type. Subregional details are available in Table S3.

**Table 1:** Area (ha) of current and potential future tidal marsh habitat, and upland areas reclaimed, under different sea-level rise and sediment availability assumptions for San Francisco Bay.

Year	Scenario	Current Land Status	Low Marsh	Mid Marsh	High Marsh	Total Marsh	Uplands Reclaimed
2010	Current	Tidal	2,992	7,572	2,464	13,029	-
2110	SSC High/SLR Low	Tidal	1,013	18,714	528	20,256	2,046
2110	SSC High/SLR High	Tidal	4,752	8,274	109	13,135	3,307
2110	SSC Low/SLR Low	Tidal	3,510	12,744	528	16,782	2,046
2110	SSC Low/SLR High	Tidal	4,422	574	109	5,104	3,307
2010	Current	Diked	3,041	3,360	1,109	7,510	-
2110	SSC High/SLR Low	Diked	5759	12,971	888	19,399	6,958
2110	SSC High/SLR High	Diked	6438	25,173	670	32,499	2,301
2110	SSC Low/SLR Low	Diked	2767	2,608	888	6,045	6,958
2110	SSC Low/SLR High	Diked	6240	10,485	670	17,613	2,301
2010	Current	Urban	1,273	1,888	1,096	4,257	-
2110	SSC High/SLR Low	Urban	3,472	10,673	1,251	15,895	13,223
2110	SSC High/SLR High	Urban	518	7,511	1,749	9,280	2,941
2110	SSC Low/SLR Low	Urban	3,883	5,692	1,251	11,325	13,223
2110	SSC Low/SLR High	Urban	1,396	4,353	1,749	6,999	2,941

To demonstrate restoration potential, the potential future marsh area for currently diked lands reflects the assumption that all barriers to inundation are removed in 2010. Suspended sediment availability (SSC) high and low assumptions vary by Bay subregion. Sea-level rise (SLR) assumptions were developed by the National Research Council (low = 0.52 m/century; high = 1.65 m/century). Values for the urban category represent areas that are considered un-restorable due to urban development.

#### Habitat change trajectories

**Figure 6:**
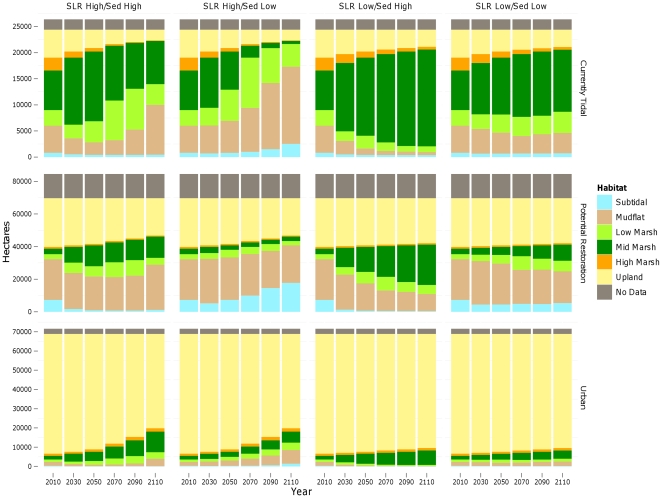
Area of potential future habitats within study area under different SLR and sediment (“Sed”) scenarios for three categories of habitat: currently tidal, potential restoration (currently diked), and urban (assumed non-restorable). Note different scales on each set of graphs.

Across most scenarios examined, intertidal habitats (mudflat through high marsh elevations) were projected to increase over the next century, reflecting the combined expansion of wetlands into current upland areas and sedimentation of currently subtidal areas. Lower rates of increase, or slight decreases, were projected toward the end of the century, as topographic limitations to marsh expansion become more important and, for the most pessimistic scenario (high SLR, low SSC), subtidal elevations increase (Fig. 6). Restoration potential for intertidal habitats (within currently diked areas) showed a similar pattern, although the area of urban development at elevations potentially subject to tidal inundation (in the absence of levees), was projected to increase even more rapidly (Fig. 6).

Only under the most optimistic scenario (low SLR, high SSC), however, was mid marsh habitat projected to continue increasing until the end of the century, both in terms of currently tidal and potential restoration areas. Under the other scenarios, mid marsh habitat was projected to increase through mid-century (2040–2080, depending on the scenario) but start declining in area thereafter. Low marsh habitats had similar projections, but would decline in existing area and increase in restoration potential under the most optimistic scenario (Fig. 6). Vegetation trajectories for potential low marsh restoration were fairly stable by the end of the century. Current areas of high marsh were projected to decrease under all scenarios, more rapidly under high rates of SLR (Fig. 6). However, restoration potential for this habitat type remained constant over time across all scenarios.

#### High marsh

**Figure 7:**
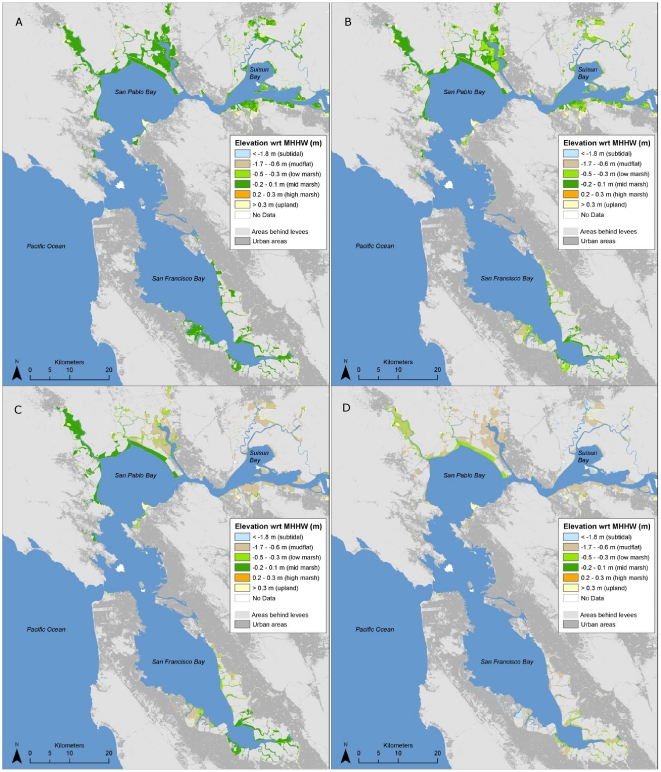
Potential 2110 intertidal habitats and elevations with respect to mean higher high water under different sea-level rise (SLR) and sediment availability assumptions with no removal of levees or other barriers to tidal inundation. (A) high sediment/low SLR, (B) low sediment/low SLR, (C) high sediment/high SLR, and (D) low sediment/high SLR. All scenarios shown assume low organic accumulation rates (1 mm/yr).

**Figure 8:**
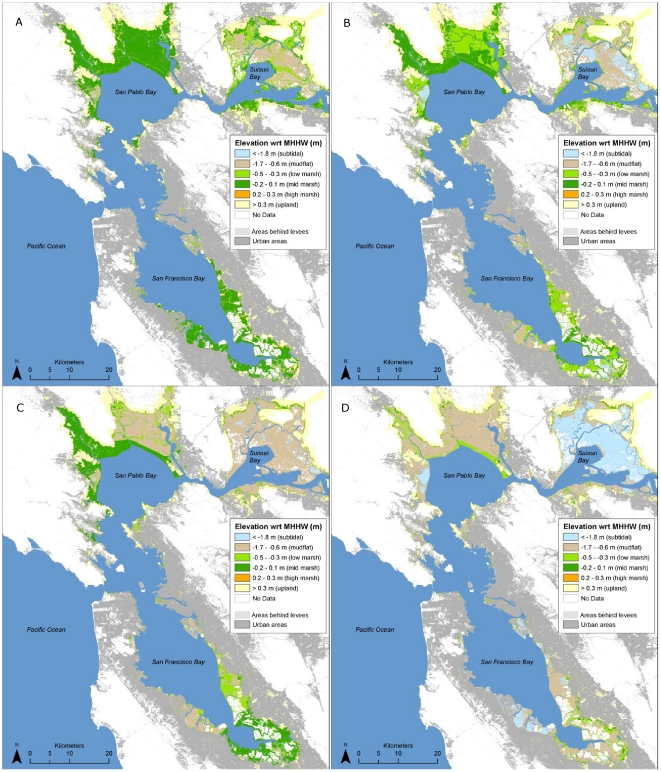
Potential 2110 intertidal habitats and elevations with respect to mean higher high water under different sea-level rise (SLR) and sediment availability assumptions with complete removal of all levees and other barriers to tidal inundation. (A) high sediment/low SLR, (B) low sediment/low SLR, (C) high sediment/high SLR, and (D) low sediment/high SLR. All scenarios shown assume low organic accumulation rates (1 mm/yr).

The area of high marsh was projected to decrease dramatically over the next century across all scenarios examined – more than any other habitat type (Table 1, Fig. 7). With a high SLR rate, the area could be reduced to just over 100 ha bay-wide by 2110; with a low rate of SLR the total projected area was just over 500 ha under both high and low SSC scenarios. While most of the future potential for this habitat would occur in areas that are already urbanized, approximately 700–900 ha are possible in undeveloped areas that are currently behind levees, dikes or roads (hereafter “diked areas”) (Table 1, Fig. 8).

#### Mid marsh

Future (100-yr) spatial habitat projections for mid marsh were highly dependent upon SSC and SLR assumptions. Under all but the most pessimistic scenario (high SLR and low SSC) the total bay-wide area of mid marsh was projected to increase to between 8,300 and 18,700 ha over the next century, as sites that are newly restored or planned for restoration in the near future (primarily former salt ponds) continue to accrete sediment and build elevation (Table 1, Fig. 7). Under the most optimistic scenario (low SLR, high SSC), 25,200 ha in currently diked areas could potentially become mid marsh habitat with new restoration efforts (Table 1, Fig. 8). However, under the more pessimistic scenario (high SLR and low SSC), the total area of mid marsh was projected to decrease dramatically, to less than 600 ha bay-wide in narrow fringes along bay margins (current upland areas). Up to 2,600 ha in currently diked upland areas (also along the bay margins) could potentially be obtained through new restoration efforts (Table 1, Fig. 8). The creation of new mid marsh habitat on up to 10,700 ha of land with potentially suitable elevations under a high rate of SLR is prevented by existing urban development (Table 1).

#### Low marsh

Low marsh habitat was projected to increase—due to a combination of mid marsh loss in some areas and new habitat creation in others—under all scenarios except for high SSC and low SLR (Table 1, Figs. 7 and 8). In this case, the decrease represented primarily a conversion to mid marsh, as low elevation areas would continue to accrete sediment.

#### Upland

The area of natural uplands projected to be reclaimed by tidal inundation (and thereby available for marsh expansion) by 2110 ranged from approximately 2,000 ha under a low rate of SLR to 3,300 ha under a high rate of SLR, as more uplands would be inundated (Table 1). Undeveloped diked uplands could provide an additional 2,300 (low SLR) to 7,000 (high SLR) ha for marsh expansion if barriers to tidal inundation were removed (Table 1). The projections for currently upland urban areas that would become tidally inundated without levee protection ranged from 2,900 (low SLR) to 13,200 (high SLR) ha.

#### Restoration potential

**Figure 9:**
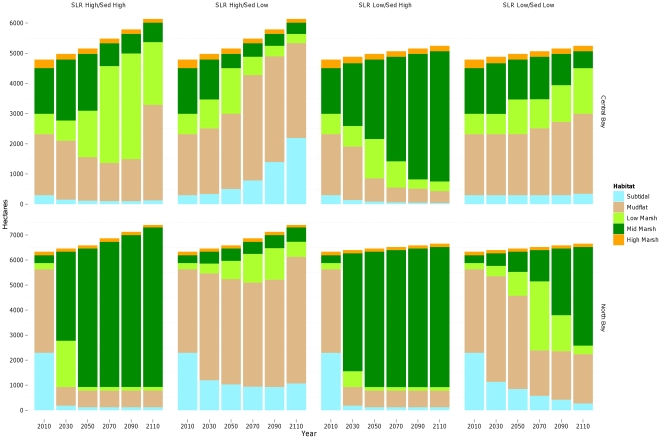
Area of potential future habitats within areas of high (North Bay) and low (Central Bay) sediment availability under different SLR and sediment (“Sed”) scenarios. Note different scales on each set of graphs.

Comparing restoration potential (for currently diked areas) between regions with low-medium sediment supply (Central Bay) and regions with high sediment supply (North Bay), future habitat trajectories were dramatically different across all scenarios examined (Fig. 9). Despite higher starting elevations, the Central Bay had lower mid marsh restoration potential than the North Bay across all scenarios. Although more mid marsh habitat could initially be restored in low sediment areas due to higher elevations (in this case), models projected an overall loss of habitat by the end of the century in all but the most optimistic scenario (Fig. 9). Conversely, the North Bay was projected to experience a net gain in mid marsh habitat by the end of the century under all scenarios.

**Figure 10:**
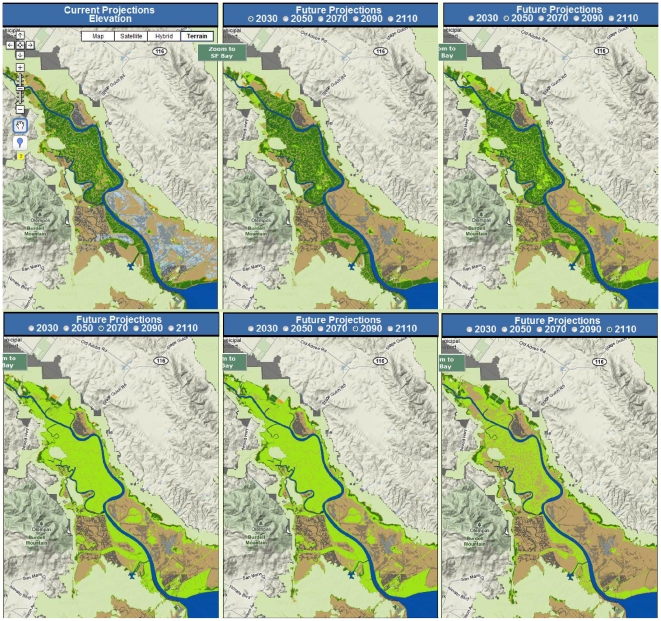
Projected elevation change for the most pessimistic scenario (low sediment, high SLR), using the on-line tool to zoom into the Petaluma River area. Maps assume an absence of levees, roads, and other barriers to tidal inundation. Maps demonstrate the increase in low and mid marsh through mid-century, followed by a decline as SLR accelerates and outpaces accretion rates. Note the limited amount of landward marsh expansion (See Table S3 for area summaries).

Under the most pessimistic scenario (high SLR, low SSC), models projected initial increases in marsh area, followed by widespread marsh drowning, with the conversion of mid marsh to low marsh in high sediment areas, as shown in an example from the Petaluma River region in the North Bay (Fig. 10), and to mudflat or subtidal habitats in low sediment areas. Projections can be further explored online (www.prbo.org/sfbayslr).

## Discussion

By applying results from a mechanistic accretion model [9] to spatial variation in sediment, salinity, and current elevations, we were able to develop spatially-explicit projections of marsh response to a set of plausible SLR scenarios for 15 San Francisco Bay subregions. When model runs were combined across subregions with different estimated SSC and OM values, Bay-wide projections of mid marsh habitat area varied substantially, depending primarily on SLR and SSC assumptions. Across all scenarios evaluated, however, our models projected a shift in the mix of intertidal habitats, with a loss of high marsh and gains in low marsh and mudflats within the study area. We found that the minimum SSC that would be required for 100-year mid marsh sustainability (i.e., no elevation loss) is greater than 300 mg/L for a high rate of SLR (1.65 m SLR/century), and between 100 and 150 mg/L for a low rate of SLR (0.5 m/century). High rates of OM accumulation had minimal impacts on this threshold in a SLR context because the maximum rate of OM accumulation that we evaluated (3 mm/year) was swamped by SLR.

Given that suspended sediment concentrations above 300 mg/L are rare in the Bay, and considering the projected acceleration of SLR beyond the 100-year timeframe examined here, our model suggests a bleak prognosis for long-term natural marsh sustainability under a high-SLR scenario. However, results also indicated that under a high rate of SLR (1.65 m/century), short-term restoration of diked subtidal baylands to mid marsh elevations (−0.2 m MHHW) within the next century could be achieved with SSC greater than 200 mg/L (100 mg/L under a low rate of SLR). Thus, even under a high-SLR scenario, opportunities for sustainable tidal marsh restoration and conservation within the next century may be found, but are limited to certain high-sediment regions of the Bay. Under a low-SLR scenario, the potential for long-term marsh sustainability and successful marsh restoration should remain high, depending on future sediment supplies.

The approach we have developed can theoretically be applied to any estuary to provide a rapid evaluation of future marsh sustainability and expansion potential. The model is an improvement on other available spatial models that predict wetland sustainability in the face of SLR because it incorporates a feedback between mineral sediment inputs and elevation [45]. Without this feedback, simple SLR projection models typically overestimate wetland loss because vertical accretion is constant at the relatively low rate that is found in high elevation, relatively mature tidal marshes. Evidence from field studies and process-based models indicates that vertical accretion rates are likely to increase in response to increases in inundation rates [7], [32], [35], [40] as long as suspended sediment concentrations are sufficient. Our model incorporates this process to create more realistic projections of marsh sustainability, which may be used to assess the vulnerability to SLR and restoration potential of individual marsh sites. An additional important contribution is the development of a user-friendly web-based mapping tool to display our results [72]. This on-line tool will allow users to compare scenarios at multiple spatial scales, to evaluate the sustainability of particular locations, and to identify potential restoration sites. Managers and decision-makers can use the tool to improve the long term effectiveness of conservation strategies by maximizing the amount of tidal marsh in high-sediment regions, identifying and prioritizing key upland transitional sites, prioritizing sediment placement, and planning for future high marsh refugia.

### Restoration and management implications

Importantly, even the most pessimistic scenario (low SSC, high SLR) resulted in projections of a Bay-wide increase in habitat until nearly 2050, indicating that large-scale effects of SLR on tidal marsh may not be seen until near the end of the century. Furthermore, due to the rapidly increasing rate of SLR projected near the turn of the next century, the trajectory of marsh loss is likely to continue at accelerated rates after 2100, with anticipated severe consequences if high rates of SLR continue. This pattern, and the potential for rapid marsh plain loss once marsh drowning begins [42] indicates the importance of proactive marsh conservation planning, via the application of sediment to raise elevations at vulnerable sites before marsh loss occurs, the prioritization of more resilient (high sediment) sites for restoration, and the protection of key upland sites as future marshland. Although our results suggest that sites with low SSC may not be sustainable regardless of starting elevation, the strategic repeated delivery of sediment could potentially be used to sustain a site indefinitely. This requires a shift in sediment management strategies to capture and redistribute excess sediment, especially clean dredge materials. Collaborative efforts to maximize the beneficial reuse of dredge materials are already underway among San Francisco Bay jurisdictions and stakeholders. Because sediment contamination is a major concern [73], [74], an approach using multiple lines of evidence to assessing sediment quality has been developed in part to inform sediment reuse decisions and minimize ecological impacts [75]. Due to regional variability in sediment availability, marsh resilience was projected to be much lower in some subregions (e.g., Central Bay) than others (e.g., North and South Bay systems). Thus, when restoration choices are explicit, efforts should be concentrated in sediment-rich areas with better prospects for long-term sustainability. However, high-vulnerability (low-sediment) subregions should be closely monitored and may provide early opportunities for validation of marsh sustainability projections. Although it would be easy to dismiss these areas, certain sites may be more amenable to intervention, and could be maintained either by restoring natural sources of sediment or by strategically applying dredge materials [71]. The relative viability of different sites would depend on factors such as wind-wave exposure, proximity to sediment sources, and accessibility, and may also be evaluated with respect to ecological values, e.g., presence of special status and endemic species.

Furthermore, future restoration priorities also should be informed by the availability of adjacent upland sites that are suitable for lateral marsh expansion or migration (i.e., undeveloped sites with very gradual slopes). Although our spatial analysis revealed relatively little area naturally available to accommodate future marshes (up to 3,300 ha under high SLR), we found that more than twice as much area (up to 7,000 ha) could be reclaimed by removing levees and other barriers to tidal action. In some of these areas, managed realignment of barriers to tidal inundation could be useful to facilitate marsh expansion while continuing to provide flood control benefits [76], [77]. Unfortunately, the large majority of areas with elevations suitable for marsh expansion within the Bay (>13,000 ha) are already urbanized and thus unavailable. The existing opportunities for marsh expansion into upland areas within particular subregions may be evaluated using our web-based tool.

### Model limitations

While we believe that the results summarized here represent the most realistic assessment currently feasible, several limitations must be emphasized. In particular, the model does not include influence of waves, which become more important as site size increases and availability of sediment diminishes [46]. Sites that are more vulnerable to waves include those with bed elevations between vegetation colonization elevation and MLLW. At these sites, wind-wave erosion may result in marsh retreat at the bay edge, and conversion of low marsh to mudflat [42]. In this respect, the projected habitat areas are most likely an overestimate of future habitat potential, especially for low marsh habitat. Conversely, future high marsh areas are likely underestimated, as we did not consider the influence of storms or other factors that may result in the deposition of new sediment above MHHW.

In addition, we had limited data from which to estimate the relative contribution of organic material to the accretion model. The organic matter calibrations were based on data from salt marshes and rates are likely higher in slightly brackish to freshwater tidal marshes. Thus, higher rates of organic accretion may currently occur,, or may occur in the future due to higher temperatures for C4 plants and higher CO_2_ concentrations for C3 plants that may increase plant productivity [12], [78]. Furthermore, the predicted increase in low marsh area would bring with it a shift in dominant species that may influence organic accretion rates resulting from different morphologies (e.g., volume of below-ground biomass) [7]. Thus, it is possible that we underestimated the potential future contribution of vegetation and organic matter inputs to marsh development, and thus future habitat potential. Additionally, although we considered decreased rates of organic accumulation as a proxy for increases in salinity that are projected to occur with SLR [79], [80], we did not explicitly consider the adverse effects of increased salinity on plant productivity and survival, which in turn could reduce the organic contribution to accretion [11]. Similarly, effects of changing inundation on organic matter processes were not included in our model.

Finally, there is some uncertainty in the range of sediment and salinity assumptions used for each subregion, as well as spatial variability within those subregions. This is especially true for more distant future time periods, given that sediment concentrations have decreased in some parts of the Bay and are likely to continue to decrease in the future [61], [80]. Although our low sediment scenario was intended to encompass such future declines, the magnitude and timing is highly uncertain. If our scenarios encompass most of this range of uncertainty, Bay-wide discrepancies are likely to be small. But for an individual site, results could change dramatically depending on actual available sediment concentrations.

### Critical future uncertainties

The large disparity across scenarios highlights the importance of future sediment supply and SLR rates in determining the fate of Bay tidal marshes. Importantly, the effects of these critical variables are not linear. There are key thresholds beyond which marshes are not sustainable, with lower rates of SLR having lower thresholds for SSC requirements.

Sediment inputs to San Francisco Bay are controlled by precipitation patterns but also upstream land use decisions and water storage and diversion practices. All of these factors have high levels of future uncertainty [81]–[83]. With the reduced precipitation that is projected for California by most general circulation models, water may become more tightly managed and thus reduce flows to the Bay, particularly during dry summer months [79], [80]. Alternatively, increased precipitation, especially when delivered by high severity storms, may bring more large pulses of fresh water and sediment to the Bay, especially during winter months. Future SLR rates are also highly uncertain, but may become more precise in the near future as models and empirical data improve.

Unfortunately these key uncertainties will be difficult to address, especially over the long term, when estimates of sediment supply and SLR become increasingly variable. In the short term, however, SLR rates can be projected with a higher level of confidence, and sediment availability can be better understood through data collection and hydrodynamic modeling. Thus, by (1) collecting better data on current suspended sediment concentrations in marshes, (2) monitoring rates of marsh accretion, and (3) proactively managing sediment within an estuary, we can improve and manipulate short-term projections of marsh sustainability. In the meantime, future SLR projections may be refined, and potentially modified via societal actions to reduce greenhouse gas emissions.

### Ecosystem ramifications

Across all scenarios evaluated, our model projections suggest a shift from high to low elevation marsh habitat, which will certainly affect vegetation composition, and will likely have cascading effects on ecological communities. The high marsh zone is high in plant diversity, relative to mid and low marsh, and hosts several endangered plant species, including soft birds-beak (*Chloropyron molle*, formerly *Cordylanthus mollis*), and many endemic species [84]. Much of this habitat has already been lost or degraded due to urban and agricultural development, restriction of tidal exchange, and the erection of levees, contributing to the endangered status of the plant and animal species that depend upon it [50], [85].

Mid marsh comprises the majority of current vegetated tidal marsh, and the primary breeding habitat of several specialized bird species, including endangered rail species, as well three endemic subspecies of tidal marsh song sparrow (*Melospiza melodia*) [86]–[88] and the endemic San Francisco common yellowthroat (*Geothlypis trichas sinuosa*) [89]. While future projections for this habitat are highly variable and dependent on sediment supply and SLR rates, its large-scale loss would have wide-reaching impacts on marsh vertebrates, which generally use low marsh to a much more limited extent (or only for foraging).

Marsh drowning will result in an increase in unvegetated intertidal habitat (i.e., mudflats), as will the inevitable erosion of low marsh habitat, especially along bay margins. This may or may not counteract expected mudflat losses within the open bay [90] but should at least provide new foraging habitats for shorebirds, waterfowl, and other waterbirds. Thus, although the loss of vegetated marsh would have negative consequences for marsh-dependent species, there are likely to be benefits for other species. As a result, restoration and conservation planning in the face of SLR will necessarily involve an evaluation of ecological trade-offs, as is already the case for current restoration planning efforts [91].

### Conclusions

Our model indicates at least two critical implications for tidal marsh habitat in the next century. First, the most optimistic scenarios for marsh habitat sustainability in the next century involve high availability of mineral sediment. However, sediment loads are physical inputs into the system that are largely controlled by upstream land use decisions and water storage and diversion practices and thus are very uncertain and likely to be dynamic over the next 100 years. Second, with high SLR and SSC less than 150 mg/L, barring the significant transfer of sediment from other areas, upland habitat will have to be captured for restoration purposes in order to make up for mid marsh habitat loss. This is a challenging scenario due to the many physical barriers currently in place that prohibit wetland migration and the complexity of land ownership surrounding the Bay.

In light of these and other challenges posed by SLR for wetland managers, realistic, spatial projections must be made available quickly and clearly to inform critical conservation prioritization and restoration planning decisions. We hope the models and results presented herein and the supporting web tool (http://www.prbo.org/sfbayslr) provide such a contribution.

## Supporting Information

Figure S1 **Data sources for mapped starting elevations within San Francisco Bay study area.**
(PDF)

Table S1 **Climate change scenario assumptions for San Francisco Bay subregions. See map in** **Figure 1** **.**
(PDF)

Table S2 **GPS-based vegetation corrections (m) used to adjust elevations in Suisun Bay marshes (subregions 14 and 15).**
(PDF)

Table S3 **Projected area (ha) of current and potential future marsh habitat, as well as upland areas reclaimed, under various sea-level rise (SLR) and sediment availability assumptions for the San Francisco Bay (Bay) estuary.** To demonstrate restoration opportunities, the potential future marsh area for currently diked lands reflects assumption that all dikes will be removed. Urban areas are not included. Suspended sediment availability (Sed) high and low assumptions vary by Bay subregion. SLR assumptions were developed by the National Research Council (low = 0.52 m/century; high = 1.65 m/century). See Table S1 for list of subregion names and sediment assumptions, and Figure 1 for subregion map.
(XLSX)
