# Supplementary figures and images for: Evaluating Tidal Marsh Sustainability in the Face of Sea-Level Rise: A Hybrid Modeling Approach Applied to San Francisco Bay
Source: PLoS One. 2011 Nov 16;6(11):e27388. doi: 10.1371/journal.pone.0027388 (PMC3217990; doi:10.1371/journal.pone.0027388)

Figure S1. Data sources for mapped starting elevations within San Francisco Bay study area.

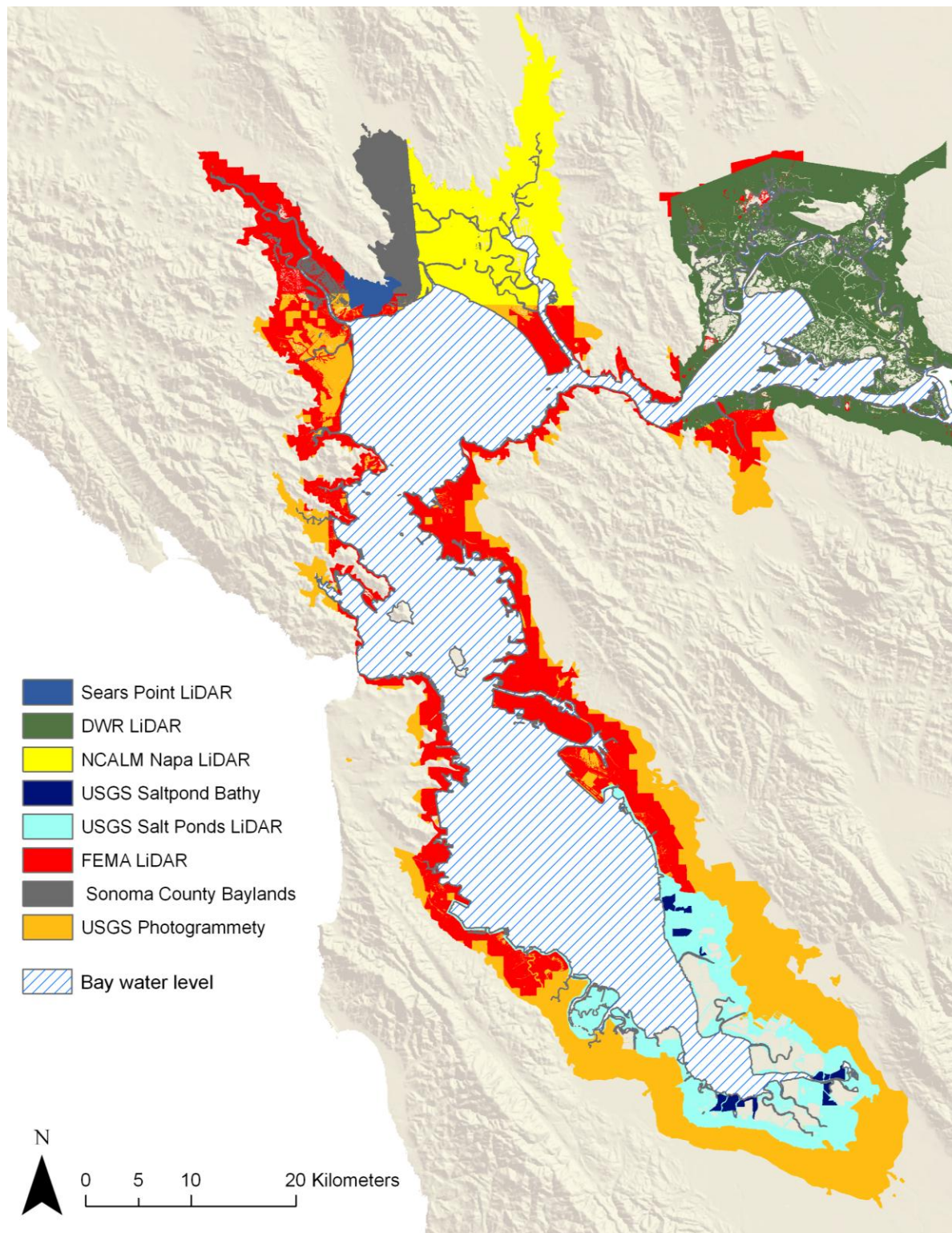

Supplement: Figure S1 — Data sources for mapped starting elevations within San Francisco Bay study area. (PDF) [file pone.0027388.s001.pdf]
