# Supplementary material for: Evaluating Tidal Marsh Sustainability in the Face of Sea-Level Rise: A Hybrid Modeling Approach Applied to San Francisco Bay
Source: PLoS One. 2011 Nov 16;6(11):e27388. doi: 10.1371/journal.pone.0027388 (PMC3217990; doi:10.1371/journal.pone.0027388)
Supplement: Table S1 — Climate change scenario assumptions for San Francisco Bay subregions. See map in Figure 1 . (PDF) [file pone.0027388.s002.pdf]

Table S1. Climate change scenario assumptions for San Francisco Bay subregions. See map in Figure 1.

		Suspended Sediment Concentration (mg/L)		Organic Material Accumulation (mm/yr)	
Area	Name	Low	High	Low	High
1	South Bay	150	300	1	1
2	Redwood City	50	150	1	1
3	Hayward	50	150	1	1
4	San Francisco	25	100	1	1
5	Oakland	50	100	1	1
6	South Marin	25	50	1	1
7	East Bay	50	100	1	1
8	North Marin	100	300	1	1
9	Pinole	50	150	1	1
10	Petaluma River	150	300	1	1
11	San Pablo North Shore	150	300	1	1
12	Napa River	100	150	1	2
13	South Suisun	150	300	2	3
14	SE Suisun	25	100	2	3
15	Suisun Marsh	25	100	2	3
