# Supplementary material for: Evaluating Tidal Marsh Sustainability in the Face of Sea-Level Rise: A Hybrid Modeling Approach Applied to San Francisco Bay
Source: PLoS One. 2011 Nov 16;6(11):e27388. doi: 10.1371/journal.pone.0027388 (PMC3217990; doi:10.1371/journal.pone.0027388)
Supplement: Table S2 — GPS-based vegetation corrections (m) used to adjust elevations in Suisun Bay marshes (subregions 14 and 15). (PDF) [file pone.0027388.s003.pdf]

Table S2. GPS-based vegetation corrections (m) used to adjust elevations in Suisun Bay marshes (subregions 14 and 15).

Suisun Marsh (subregion 15)	
Schoenoplectus americanus/Potentilla spp.	-0.33
Typha angustifolia/S. americanus	-0.29
Typha spp.	-0.84
Distichlis/S. americanus	-0.11
S. californicus/S. acutus)-Typha spp.	-0.26

Brown's Island (subregion 16)	
Schoenoplectus acutus/S. californicus	-0.63
Typha spp.	-0.60
S. americanus	-0.60
Phragmities australis	-0.83
Rubus discolour	-0.98
